# Defensive medicine in primary health care

**DOI:** 10.1080/02813432.2018.1499483

**Published:** 2018-08-08

**Authors:** Jesper Lykkegaard, Merethe Kirstine Kousgaard Andersen, Jørgen Nexøe, Elisabeth Assing Hvidt

**Affiliations:** a Research Unit of General Practice, Institute of Public Health, University of Southern Denmark, Odense, Denmark;; b Editorial Board, Scandinavian Journal of Primary Health Care

During the past year, a case of a young Danish doctor in vocational training as general practitioner accused in court of gross negligence has been all over the Danish media. A patient died maybe because of over dosage of insulin. The doctor’s ordering of blood glucose measurement was given verbally but not written in the patient record and the normal procedures for a patient with diabetes failed after the doctor had left her night shift. The young doctor was first acquitted of all charges in primary court, then convicted in national court, and finally cleared in Supreme Court by a three judges versus two ruling.

During the process, thousands of Danish doctors, including many in primary health care, joined the hashtag campaign #DetKuHaVaeretMig (#ItCouldHaveBeenMe) in support of the doctor accused and as a way of protesting against perceived misconduct of the Danish Patient Safety Authority (STPS) as well as the hospital management.

It is worrying that the case ended in the judicial system, despite the fact that the doctor's actions were not significantly different from ordinary practice. Stories like the above increase the fear of malpractice claims and police accusations among doctors. Doctoring seems to be hampered by a harmful zero-mistake culture that is likely to have enormous consequences for patients and doctors as well as society.

Defensive medicine (DM) refers to actions that healthcare providers take in order to protect themselves from malpractice claims rather than actions benefitting the patient. DM has been demonstrated in healthcare systems all over the world and is documented to have increased during the past few years. For example, DM has been estimated to account for 10% of all spent healthcare resources in Italy [1]. It affects peoples’ lives from even before birth, in the way that obstetricians’ choice of making a C-section is motivated by defensive behaviour [2].

In a recent Danish study among GPs DM was experienced on a daily basis as actions taken because of pressures deriving from four different sources: “the system,” the patients, the GPs themselves and their peers [3]. In particular, the system-imposed pressure to document every medical action in detail was experienced as leading to meaningless and even potentially harmful doctoring.

The hashtag campaign #DetKuHaVaeretMig is about clearer rules for how detailed medical actions need to be recorded. Detailed patient records may protect doctors from being blamed in case of a patient complaint and hence from becoming second victims. Older GPs make less detailed patient records. Maybe therefore they are more likely to be disciplined in case of a complaint compared to younger [4]. This practice and stories like the young doctor’s enforces a tendency towards automatized, fully detailed, all covering, and knowingly redundant patient records. However, long patient records and computer-generated journal phrases may do well in a juridical setting at the risk of making the record a less useful in the clinical setting, increasing the risk of overlooking important information, slowing down GPs’ work and reducing the time spent with the patients. It favours strategic and cynical doctor-patient interaction leading to low job satisfaction and preterm retirement [5].

It is time to confront and reduce the pressures for meaningless doctoring rather than continue adding longer phrases to the computer systems.
